# First-in-human study of GFH018, a small molecule inhibitor of transforming growth factor-β receptor I inhibitor, in patients with advanced solid tumors

**DOI:** 10.1186/s12885-024-12216-7

**Published:** 2024-04-10

**Authors:** Ye Guo, Zishu Wang, Huan Zhou, Hongming Pan, Weidong Han, Yanhong Deng, Qun Li, Junli Xue, Xiaoxiao Ge, Shuang Wang, Jing Wang, Yue Zhang, Congqiao Zhao, Huaqiang Zhu, Yu Wang, Haige Shen, Dong Liu, Jin Li

**Affiliations:** 1 Department of Medical Oncology, Shanghai East Hospital, School of Medicine, Tongji University, Shanghai, China; 2 The First Affiliated Hospital of Bengbu Medical College, Bengbu, China; 3 Department of Medical Oncology, Sir Run Run Shaw Hospital, Zhejiang University School of Medicine, Hangzhou, Zhejiang China; 4 Sixth Affiliated Hospital of Sun Yat-Sen University, Guangdong, China; 5 Clinical Department, GenFleet Therapeutics Inc, Shanghai, China; 6 Translational Science, GenFleet Therapeutics Inc, Shanghai, China

**Keywords:** Transforming growth factor-β, Immunomodulation, Tumor Microenvironment, Tumor Biomarkers

## Abstract

**Background:**

Transforming growth factor-β (TGF-β) is a cytokine with multiple functions, including cell growth regulation, extracellular matrix production, angiogenesis homeostasis adjustment and et al. TGF-β pathway activation promotes tumor metastasis/progression and mediates epithelial-mesenchymal transmission suppressing immunosurveillance in advanced tumors. GFH018, a small molecule inhibitor blocking TGF-β signal transduction, inhibits the progression and/or metastasis of advanced cancers. This first-in-human study evaluated the safety, tolerability, pharmacokinetics (PK), and efficacy of GFH018 monotherapy in patients with advanced solid tumors.

**Methods:**

This phase I, open-label, multicenter study used a modified 3+3 dose escalation and expansion design. Adult patients with advanced solid tumors failing the standard of care were enrolled. Starting at 5 mg, eight dose levels up to 85 mg were evaluated. Patients received GFH018 BID (14d-on/14d-off) starting on the 4th day after a single dose on cycle 1, day 1. Subsequent cycles were defined as 28 days. The study also explored the safety of 85 mg BID 7d-on/7d-off. Adverse events were graded using NCI criteria for adverse events (NCI-CTCAE v5.0). PK was analyzed using a noncompartmental method. Efficacy was evaluated using RECIST 1.1. Blood samples were collected for biomarker analysis.

**Results:**

Fifty patients were enrolled and received at least one dose of GFH018. No dose-limiting toxicity occurred, and the maximum tolerated dose was not reached. Forty-three patients (86.0%) had at least one treatment-related adverse event (TRAE), and three patients (6.0%) had ≥ G3 TRAEs. The most common TRAEs (any grade/grade ≥3) were AST increased (18%/0%), proteinuria (14%/2%), anemia (14%/2%), and ALT increased (12%/0%). No significant cardiotoxicity or bleeding was observed. GFH018 PK was linear and dose-independent, with a mean half-life of 2.25–8.60 h from 5 – 85 mg. Nine patients (18.0%) achieved stable disease, and one patient with thymic carcinoma achieved tumor shrinkage, with the maximum target lesion decreased by 18.4%. Serum TGF-β1 levels were not associated with clinical responses. The comprehensive recommended dose for Phase II was defined as 85 mg BID 14d-on/14d-off.

**Conclusions:**

GFH018 monotherapy presented a favorable safety profile without cardiac toxicity or bleeding. Modest efficacy warrants further studies, including combination strategies.

**Trial registration:**

ClinicalTrial. gov (https://www.clinicaltrials.gov/), NCT05051241. Registered on 2021-09-02.

**Supplementary Information:**

The online version contains supplementary material available at 10.1186/s12885-024-12216-7.

## Background

Transforming growth factor-β (TGF-β) is a pleiotropic cytokine that regulates embryogenesis and tissue homeostasis by signaling cascades [1, 2]. By binding to TGF-β receptor II, TGF-β induces the phosphorylation of TGF-β receptor I inhibitor (TGF-βRI) and SMAD2/3, modulating gene expression and physiological functions [1, 3]. The actual reaction is modulated by the cell context and correlates with other signaling pathways [4]. Among the three TGF-β isoforms, TGF-β1 predominates in the tumor microenvironment (TME) [5]. It plays a dual role, acting as both a tumor suppressor and promoter of tumor metastasis, depending on the cancer stage. Numerous studies have shown that in advanced cancer, overexpression of TGF-β leads to epithelial-mesenchymal transition of tumor cells, angiogenesis in the TME, and tissue fibrogenesis, exhibiting a tumor-promoting function, which is distinct from the early stage [3].

Emerging evidence indicates that TGF-β signaling promotes resistance to therapies (chemotherapy, targeted therapy, and immunotherapy) [6]. This pathway may be leveraged to reshape the immunosuppressive TME and define a bypass mechanism for immune checkpoint inhibitor therapy [7, 8]. Preclinical and clinical findings show that TGF-β blockade improves the anti-PD-1/L1 response [9, 10].

Given its crucial role, the TGF-β signaling pathway has emerged as a prominent target for cancer therapy, and several types of inhibitors have been developed, but none have been approved by an authority to treat any cancer type yet [11, 12]. GFH018 is a novel small molecular inhibitor (SMI) of TGF-βRI that blocks TGF-β signal transduction, inhibiting the progression and metastasis of advanced cancers. Preclinical evidence has shown its ability to inhibit TGF-β-induced SMAD3 phosphorylation and regulate the TME [13]. Synergistic effects were observed in combination with anti-PD-1/L1 antibodies [13]. This first-in-human (FIH) study of GFH018 was aimed at assessing the safety/tolerability across different dose levels and determining the recommended dose for expansion (RDE) and/or recommended phase 2 dose (RP2D). Preliminary efficacy was also explored. Herein, we report the results of the completed GFH018 FIH study.

## Methods

### Patient population

The study was conducted at five sites in China, and the site list is provided in the Supplementary Material. Eligible patients were aged 18–75 years with Eastern Cooperative Oncology Group Performance Status (ECOG P.S. ) ≤ 1 and had adequate cardiovascular, liver, and renal functions. For the escalation part, patients with nonmeasurable lesions according to the response evaluation criteria in solid tumors (RECIST) 1.1 were included, while for expansion, patients had to have at least one measurable lesion. The full inclusion and exclusion criteria are listed in the Supplementary Material.

This study was conducted in accordance with the Declaration of Helsinki and the Good Clinical Practice guidelines of the International Council for Harmonization. The local Ethics Committee reviewed and approved the study protocol (Online Supplementary Table S1), and each patient provided written informed consent prior to the study procedure.

### Study design and procedure

This was a phase I, open-label, multicenter study (NCT#05051241) of GFH018 in patients with advanced solid tumors who had failed the standard therapies. The study consisted of a dose-escalation part followed by an expansion part. In the dose escalation part, a 3-day pharmacokinetic (PK) lead-in period was conducted for a single dose in the first treatment cycle. Patients received GFH018 once on cycle 1 day 1 (C1D1) and underwent serial PK testing over the first 3 days. On C1D4, patients were administered the corresponding dose of GFH018 BID 14 days on/14 days off. The first cycle was defined as 31 days, including 3-day PK leading-in period with GFH018 single dosing and then 14d-on/14d-off dosing schedule. The subsequent cycles were 28 days. The starting dose of GFH018 was 5 mg, with eight planned dose levels up to 85 mg (5, 10, 20, 30, 40, 50, 65, and 85 mg). A modified 3+3 design was used to determine the maximum tolerance dose (MTD) and RDE, guided by safety/tolerability and PK data. Dose-limiting toxicities (DLTs) were evaluated during the first cycle at each dose level. The safety of 85 mg BID, 7 d-on/7 d-off, was explored after confirming the safety of 14 d-on/14 d-off. The DLTs were defined as hematological toxicities, including G4 neutropenia lasting for more than 5 days, G3 febrile neutropenia, G4 anemia, G4 thrombocytopenia, or G3 thrombocytopenia accompanied by bleeding, and ≥ G3 nonhematological toxicity (except for diarrhea, nausea, vomiting, and rash recovering ≤ G2 after supportive treatment within 3 days). The DLTs for cardiac toxicities included ≥ G2 cardiac valve abnormalities, ≥ G2 left ventricular ejection fraction (LVEF) decrease, any cardiovascular impairment shown in imaging, and abnormal highly sensitive troponin (hs-Tn) increased to ≥ twice the baseline in two consecutive tests with an interval ≥ 3 days. In the expansion part, patients were orally administered GFH018 RDE twice daily to further evaluate the safety of RDE and other dose regimens. Several types of tumors were predefined based on the biological mechanism of expansion, including nasopharyngeal carcinoma (NPC), biliary tract carcinoma (BTC), and head and neck squamous cell carcinoma (HNSCC). The treatment cycle lasted 28 days. A comprehensive RP2D would be determined based on the data totality.

### Safety

All enrolled patients were monitored regularly after GFH018 administration. Safety assessments included adverse events (AEs), serious AEs (SAEs), laboratory assessments, vital signs, physical examinations, electrocardiography and echocardiography. AEs were graded according to the NCI Common Terminology Criteria for Adverse Events (NCI-CTCAE) v5.0. All AEs were followed up until they were stable or had recovered to baseline. Adverse events of special interest (AESIs) were predefined as echocardiographic abnormalities such as aggravated stenosis or regurgitation of the heart valves, clinically significant decreases in LVEF, brain natriuretic peptide (BNP) or N-terminal pro-brain natriuretic peptide (NT-proBNP) increases, and hs-Tn increases judged by investigators.

### Pharmacokinetics

PK analysis included patients who received at least one dose of GFH018 and had measurable plasma concentrations. For patients enrolled in the escalation part of the study, PK blood samples were collected at C1D1 predose; 0.5 h, 1 h, 2 h, 4 h, 8 h, and 12 h postdose; d 2 (24 h), d 3 (48 h), d 4 (72 h), and d 10 predose; d 17 (for 14 d-on/14 d-off regimen) or d 24 (for 7 d-on/7 d-off regimen) predose; and 0.5 h, 1 h, 2 h, 4 h, 8 h, and 12 h postdose. For patients enrolled in the expansion part, PK samples were collected predose on the last administration day and 0.5 h, 1 h, 2 h, 4 h, 8 h, and 12 h postdose on the last administration day. GFH018 levels in plasma samples were analyzed using a validated liquid chromatography-tandem mass spectrometry method. GFH018 PK parameters were determined using noncompartmental analysis methods and calculated using Phoenix WinNonlin Version 8.3.1 (Certara, Princeton, NJ, USA). GFH018 plasma concentrations and PK parameters were summarized descriptively according to the dose level.

### Biomarker assessment

Serum samples were collected at baseline, 1 h after the first dosing on C1D1, and 1 h after the last dose of cycles 1, 4, 7, and 10. Serum TGF-β1 levels were analyzed using an enzyme-linked immunosorbent assay (R&D Systems, Cat #DB100B).

Peripheral blood mononuclear cells (PBMCs) were isolated from whole blood samples for pharmacodynamic assessment. Phosphorylated SMAD2 (pSMAD2) in PBMCs was measured using the AlphaLISA® SureFire® Ultra™ assay (Perkin Elmer, Cat #ALSU-PSM2-A-HV).

### Clinical efficacy

The endpoints for efficacy, including the objective response rate (ORR), disease control rate (DCR), progression-free survival (PFS), and time to progression (TTP), were assessed per RECIST 1.1. Tumor response was evaluated every two treatment cycles until disease progression, start of a new antitumor treatment, consent withdrawal, loss to follow-up, or study termination for other reasons.

### Statistical analysis

For the safety and efficacy analyses, data from patients who received GFH018 at the same dose in the dose-escalation and expansion cohorts were pooled. The demographic and baseline characteristics were summarized using descriptive methods. Safety and efficacy data were summarized for all patients who received at least one dose of GFH018. The ORR and DCR were summarized by cohort and overall. When the sample size of the analyzed group was ten or more, the Kaplan–Meier method was used to estimate the median PFS and TTP. In this study, treatment-emergent AEs (TEAEs) were defined as AEs that occurred on or after the first dosing date of GFH018 and no later than 30 days after the last dose of GFH018. The incidence and severity of AEs were descriptively summarized according to dose level. The PK parameters of GFH018 were calculated using noncompartmental analysis methods. All plasma PK data were summarized by cohort and visit day or time using descriptive statistics.

## Results

### Patient demographics and baseline characteristics

**Fig. 1:**
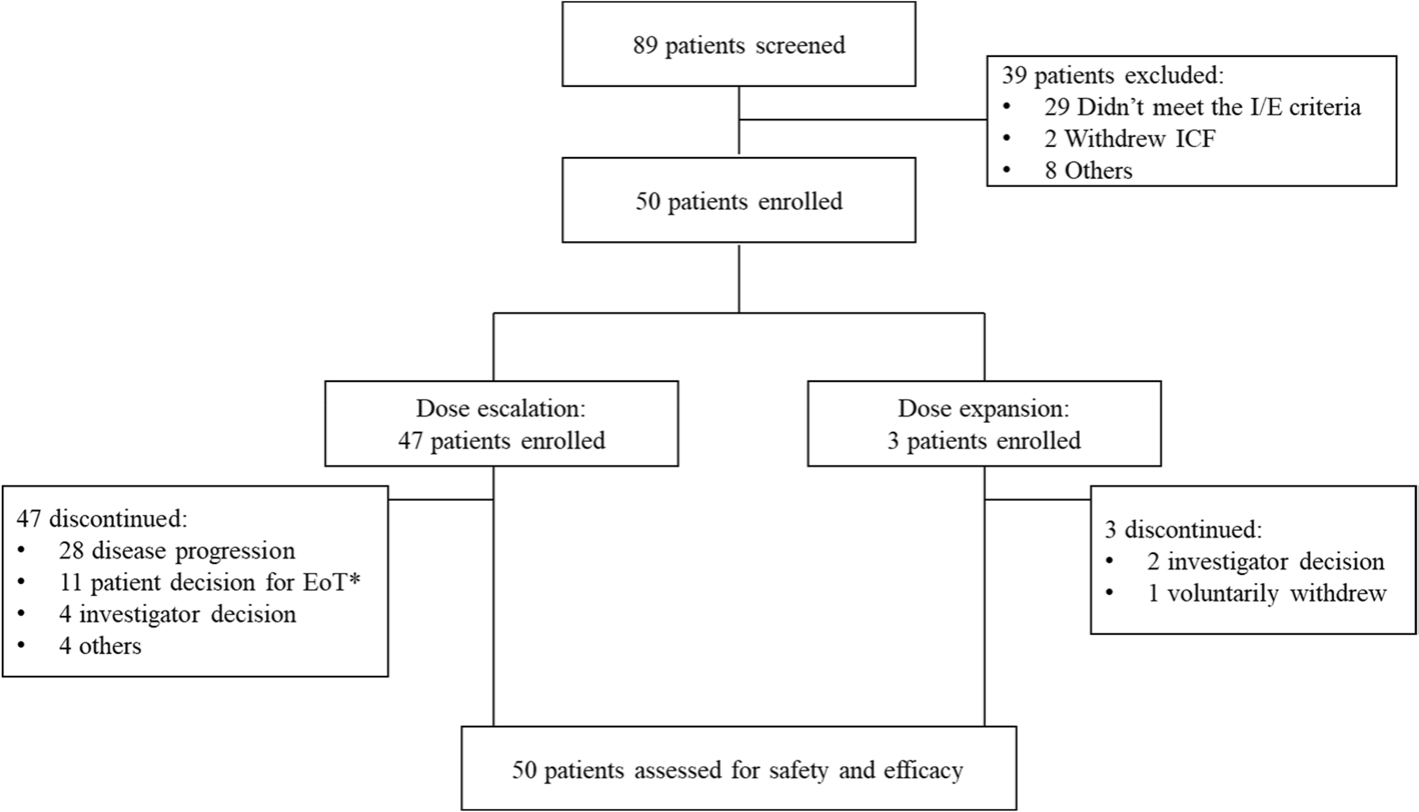
Patient distribution. Abbreviation: EoT, end of treatment. *A total of 11 patients decided to discontinue the study treatment but were still kept in the study for disease progression and/or safety follow up

In total, eighty-nine patients were screened, among whom 50 patients (14 d-on/14 d-off: 5 mg [*n*=4], 10 mg [n=3], 20 mg [*n*=4], 30 mg [*n*=7], 40 mg [*n*=4], 50 mg [*n*=4], 65 mg [*n*=6] and 85 mg [*n*=12]; 7 d-on/7 d-off: 85 mg [*n*=6]) were enrolled and received GFH018 treatment as single agent from August 2019 to August 2022. The median duration of drug exposure for all patients was 36.5 days (range, 5–213 days). The patient distribution is shown in Fig. 1. All 50 patients discontinued the study, with most patients discontinuing the study due to progressive disease (56.0%) and patient decision for end of treatment (22.0%).

The median age of the 50 enrolled patients was 52.5 years (range, 27–68 years). A wide range of solid tumors was observed, with colorectal cancer being the most common (13 patients, 26.0%), followed by non-small cell lung cancer (NSCLC, 8 patients, 16.0%), gastric and esophageal cancers, and endometrial cancer (3 patients, 6.0% each). Forty-seven patients (94.0%) had ECOG scores of 1. Regarding prior anti-tumor history, 37 (74.0%) patients had ≥3 prior systemic treatment lines, and 24 patients (48.0%) had progressed prior to PD-1/L1 therapies. Similar baseline demographic characteristics were observed at each dose level. The baseline characteristics of the enrolled patients are summarized in Table 1.

**Table 1:** Patient demographics and baseline characteristics

		**Total** **(*N* = 50)**
**Age, y**	Median (Min, max)	52.5 (27, 68)
**Sex**	Male, *n* (%)	23 (46.0%)
**ECOG, *n* (%)**	0	3 (6.0%)
	1	47 (94.0%)
**Tumor types**	CRC	13 (26.0%)
	NSCLC	8 (16.0%)
	GC/GEJC	3 (6.0%)
	Endometrial cancer	3 (6.0%)
	Breast cancer	2 (4.0%)
	Others	21 (42.0%)
**Prior antitumor** **Regimen, *n* (%)**	0	0
	1	5 (10.0%)
	2	7 (14.0%)
	≥ 3	37 (74.0%)
**Prior anti-PD-1 therapy**	Yes	24 (48.0%)
	No	26 (52.0%)

*Abbreviations: NSCLC* non-small cell lung cancer, *GC* gastric cancer, *GEJC* gastroesophageal junction cancer

### Safety/tolerability

Approximately 48 patients (96.0%) experienced at least one TEAE, and 13 (26.0%) experienced at least one ≥ G3 TEAE. Treatment discontinuation occurred in two patients due to bilirubin increased and peripheral edema, none of which were related to GFH018, as judged by the investigator. Forty-three patients (86.0%) had at least one treatment-related adverse event (TRAE), and three patients (6.0%) were reported to have ≥ G3 TRAEs, which included anemia, proteinuria, lymphocyte count decrease, hypocalcemia, and blood creatine phosphokinase increase. No grade 4 or 5 TRAEs were observed, no DLTs were observed at any dose level, and the MTD was not reached. TEAEs with an incidence ≥ 10% are shown in Table S2. The most common TRAEs (all G/≥G3, Table 2) were AST increased (18.0%/0), proteinuria (14.0%/2.0%), anemia (14.0%/2.0%), ALT increased (12.0%/0), lymphocyte count decreased (12.0%/2.0%), urine protein present (12.0%/0), GGT increased, ALP increased, and LDH increased (all 10.0%/0). No bleeding events occurred. During the study, nine patients experienced at least one serious AE (SAE), none of which were related to GFH018. Six deaths were reported, all of which were judged by the investigators to be unrelated to GFH018. No significant cardiotoxicity was observed, and all AESIs were mild (Grade 1) without any clinical symptoms or signs.

**Table 2:** Treatment-related adverse events with incidence ≥ 10%

**Preferred term**	**GFH018** **5 mg BID** **14d-on/14d-off** **(*N* = 4)** ***n* (%)**	**GFH018** **10 mg BID** **14d-on/14d-off** **(*N* = 3)** ***n* (%)**	**GFH018** **20 mg BID** **14d-on/14d-off** **(*N* = 4)** ***n* (%)**	**GFH018** **30 mg BID** **14d-on/14d-off** **(*N* = 7)** ***n* (%)**	**GFH018** **40 mg BID** **14d-on/14d-off** **(*N* = 4)** ***n* (%)**	**GFH018** **50 mg BID** **14d-on/14d-off** **(*N* = 4)** ***n* (%)**	**GFH018** **65 mg BID** **14d-on/14d-off** **(*N* = 6)** ***n* (%)**	**GFH018** **85 mg BID** **7d-on/7d-off** **(*N* = 6)** ***n* (%)**	**GFH018** **85 mg BID** **14d-on/14d-off** **(*N* = 12)** ***n* (%)**	**Total** **(*N* = 50)** ***n* (%)**
**At least one TRAE**	**4 (100%)**	**3 (100%)**	**4 (100%)**	**7 (100%)**	**4 (100%)**	**2 (50.0%)**	**5 (83.3%)**	**3 (50.0%)**	**11 (91.7%)**	**43 (86.0%)**
AST increased	0	0	0	2 (28.6%)	0	1 (25.0%)	2 (33.3%)	0	4 (33.3%)	9 (18.0%)
Proteinuria	0	0	2 (50.0%)	3 (42.9%)	0	0	1 (16.7%)	0	1 (8.3%)	7 (14.0%)
Anemia	0	1 (33.3%)	0	0	0	0	2 (33.3%)	1 (16.7%)	3 (25.0%)	7 (14.0%)
Protein urine present	1 (25.0%)	1 (33.3%)	1 (25.0%)	0	0	1 (25.0%)	0	1 (16.7%)	1 (8.3%)	6 (12.0%)
Lymphocyte count decreased	1 (25.0%)	0	0	1 (14.3%)	0	0	0	2 (33.3%)	2 (16.7%)	6 (12.0%)
ALT increased	0	1 (33.3%)	0	2 (28.6%)	0	1 (25.0%)	0	0	2 (16.7%)	6 (12.0%)
GGT increased	0	2 (66.7%)	0	0	0	1 (25.0%)	0	0	2 (16.7%)	5 (10.0%)
ALP increased	0	1 (33.3%)	0	0	1 (25.0%)	1 (25.0%)	0	1 (16.7%)	1 (8.3%)	5 (10.0%)
LDH increased	0	1 (33.3%)	0	1 (14.3%)	1 (25.0%)	0	0	0	2 (16.7%)	5 (10.0%)

Data are shown as *n* (%). Adverse events were coded per MedDRA 25.0 and graded according to CTCAE 5.0

*Abbreviations: TRAEs* treatment-related adverse events, *ALT* alanine aminotransferase, *AST* aspartate aminotransferase, *GGT* γ-glutamyl transpeptidase, *ALP* alkaline phosphatase, *LDH* lactate dehydrogenase

The safety profile in patients treated with the 7 d-on/7 d-off regimen was not significantly different to that observed in patients treated with the 14 d-on/14 d-off regimen. Higher incidences of ALT/AST and GGT increased were observed in the 14d-on/14d-off than 7d-on/7d-off. However, all of them were Grade 1 or 2 per CTCAE (see Table S3).

### Pharmacokinetics

GFH018 was rapidly absorbed after oral administration. The median T_max_ following single dosing was approximately consistent across cohorts ranging from 0.49 to 0.96 h. The geometric mean t_1/2_ ranged from 3.11 to 8.60 h. Plasma exposure to GFH018 (AUC_0-t_ and C_max_) increased in an approximately proportional manner within the dose range of 5–85 mg.

**Fig. 2:**
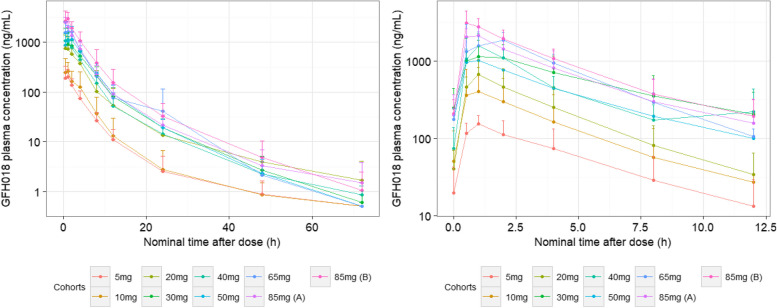
Mean (+SD) plasma concentration-time profiles of GFH018 (left panel: single dose; right panel: multiple dose). Cohort 85mg **A** represents 85 mg BID 7d-on/7d-off regimen. Cohort 85mg **B** represents 85 mg BID 14d-on/14d-off regimen

Following administration of multiple doses for 7 or 14 d, a steady-state concentration of GFH018 was reached. The median T_max_ at the steady state ranged from 0.5 to 1.8 h. The geometric mean of t_1/2_ ranged from 2.25 to 4.09 h, which did not correlate with the dose level or dosing regimen. The PK profiles after multiple doses were similar to those observed after a single dose. Similar to single dosing, exposure to GFH018 increased in an approximately proportional manner in the dose range of 5–85 mg. No obvious accumulation of GFH018 was observed in any cohort. The geometric mean of the accumulation ratio calculated using the AUC and R_acc_ across all dose groups was 1.02–1.32. A summary of PK concentrations can be found in Table 3. For the PK profile of GFH018, please refer to Fig. 2.

**Table 3:** Summary of GFH018 PK parameters

	**GFH018** **5 mg BID** **14d-on/14d-off**	**GFH018** **10 mg BID** **14d-on/14d-off**	**GFH018** **20 mg BID** **14d-on/14d-off**	**GFH018** **30 mg BID** **14d-on/14d-off**	**GFH018** **40 mg BID** **14d-on/14d-off**	**GFH018** **50 mg BID** **14d-on/14d-off**	**GFH018** **65 mg BID** **14d-on/14d-off**	**GFH018** **85 mg BID** **7d-on/7d-off**	**GFH018** **85 mg BID** **14d-on/14d-off**
**Single dose**	**(*N* = 4)**	**(*N* = 3)**	**(*N* = 4)**	**(*N* = 7)**	**(*N* = 4)**	**(*N* = 4)**	**(*N* = 6)**	**(*N* = 6)**	**(*N* = 9)**^**a**^
C_max_ (ng/mL)	215 (50.2)	250 (72.2)	743 (65.1)	972 (82.7)	1180 (24.7)	1209 (85.4)	2172 (16.3)	2555 (29.9)	3225 (35.5)
T_max_ (h)	0.65 (0.40, 0.97)	0.88 (0.42, 0.93)	0.49 (0.43, 1.03)	0.95 (0.37, 3.80)	0.69 (0.37, 0.90)	0.96 (0.47, 1.92)	0.68 (0.48, 2.00)	0.50 (0.50, 1.10)	0.58 (0.45, 1.12)
AUC_0-12_ (h*ng/mL)	690 (51.3)	819 (99.0)	2551 (107.3)	4250 (50.4)	4147 (30.3)	5100 (57.9)	7028 (10.9)	7455 (27.3)	10074 (42.0)
AUC_0-t_ (h*ng/mL)	754 (58.4)	852 (111.3)	2802 (123.7)	4895 (48.2)	4561 (35.3)	5858 (50.2)	7831 (17.8)	8240 (27.9)	11119 (45.5)
AUC_0-inf_ (h*ng/mL)	769 (58.3)	864 (110.5)	2825 (123.9)	4926 (47.8)	4603 (35.2)	5906 (49.3)	7870 (17.9)	8284 (28.1)	11164 (45.4)
t_1/2_ (h)	4.42 (82.9)	3.11 (106.3)	4.90 (115.1)	5.74 (37.6)	8.60 (103.8)^b^	5.93 (51.2)	5.51 (34.3)	7.45 (61.8)	6.37 (41.6)
**Multiple dose**	**(*N*=3)**	**(*N*=3)**	**(*N*=3)**	**(*N*=7)**	**(*N*=3)**	**(*N*=2)**	**(*N*=6)**	**(*N*=5)**	**(*N*=7)**^**c**^
C_max_ (ng/mL)	165 (15.7)	291 (124.9)	676 (48.0)	1167 (87.9)	1599 (33.7)	1253 (7.3)	2303 (34.0)	2193 (29.1)	3034 (33.6)
T_max_ (h)	0.88 (0.42, 0.95)	0.92 (0.90, 1.27)	0.93 (0.57, 1.08)	1.10 (0.47, 2.58)	0.88 (0.38, 1.00)	0.75 (0.50, 1.00)	1.88 (0.50, 2.00)	0.50 (0.50, 1.00)	0.50 (0.50, 2.05)
t_1/2_ (h)	2.67 (31.0)	2.25 (26.5)	2.55 (24.6)	4.09 (36.8)	2.45 (42.8)	3.53 (3.1)	2.59 (9.6)	2.92 (18.3)	3.01 (20.0)^d^
C_min_ (ng/mL)	7.48 (208.2)	15.8 (212.1)	25.5 (137.3)	144 (107.2)	21.0 (2741.3)	91.4 (72.9)	129 (51.8)	115 (92.5)	164 (54.3)
AUC_tau_ (h*ng/mL)	597 (61.1)	997 (168.9)	2056 (76.9)	5605 (76.6)	5144 (30.3)	4432 (43.6)	8189 (29.3)	7732 (46.8)	11353 (27.9)
R_acc_	1.02 (37.9)	1.22 (40.3)	1.15 (17.4)	1.32 (39.4)	1.18 (6.7)	1.26 (9.2)	1.17 (25.3)	1.04 (32.5)	1.18 (24.1)^e^

The parameters are presented as the geometric mean (geometric CV), and T_max_ is presented as the median (minimum, maximum)

*Abbreviations: C*_*max*_ maximum concentration, *T*_*max*_ time to reach maximum concentration, *AUC*_*0-12*_ area under the curve from time zero to 12 h, *AUC*_*0-t*_ area under the curve from time zero to the time of the last quantifiable concentration, *AUC*_*0-inf*_ area under the curve from time zero to infinity, *t*_*1/2*_ elimination half-life, *C*_*min*_ minimum concentration, *AUC*_*tau*_ area under the curve over a dosing interval, *R*_*acc*_ accumulation ratio calculated by AUC

^a^This cohort is only 85 mg BID 14d-on/14d-off in the dose escalation part. PK samples after a single administration were not collected in the dose expansion part

^b^The t_1/2_ of one subject in 40 mg BID 14d-on/14d-off was not reported due to R^2^ <0.75, *n*=3

^c^This cohort represents 85 mg BID 14d-on/14d-off in the dose escalation part combined with 85 mg BID 14d-on/14d-off in the dose expansion part

^d^The t_1/2_ of two subjects in 85 mg BID 14d-on/14d-off in the dose expansion part was not reported due to R^2^ <0.75, *n*=5

^e^PK samples after a single administration were not collected in the dose expansion part. Therefore, the R_acc_ of these two subjects cannot be calculated, *n*=5

### Clinical efficacy

**Fig. 3:**
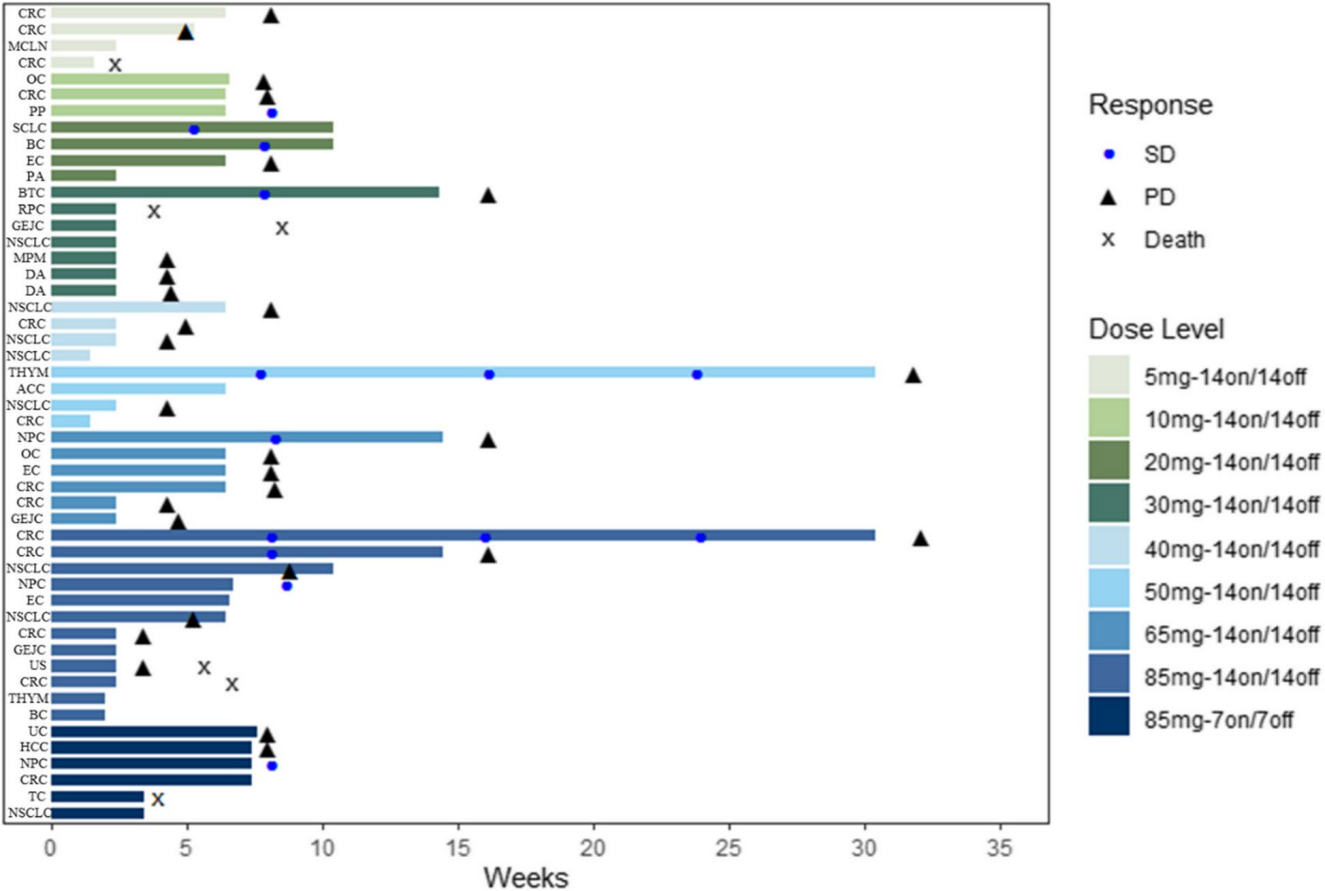
Exposure duration and overall response. Abbreviations: SD, stable disease. PD, progressive disease. ACC, adenoid cystic carcinoma. BC, breast cancer. BTC, biliary tract cancer. CRC, colorectal cancer. DA, duodenal adenocarcinoma. EC, endometrial carcinoma. GEJC, gastroesophageal junction carcinoma. MCLN, metastatic carcinoma of lymph nodes. MPM, malignant pleural mesothelioma. NSCLC, non-small cell lung cancer. NPC, nasopharyngeal carcinoma. OC, ovarian cancer. PA, pancreatic adenocarcinoma. PP, pelvic paraganglioma. RPC, renal pelvis carcinoma. SCLC, small cell lung cancer. TC, thyroid carcinoma. THYM, thymic carcinoma. UC, urachus carcinoma. US, uterus sarcoma

Out of the 50 enrolled patients, 33 underwent at least one tumor assessment after receiving treatment. No complete response (CR) or partial response (PR) was observed. Nine patients (18.0%) treated with doses ranging from 10 to 85 mg BID achieved stable disease (SD). Among these patients, one with thymic carcinoma receiving 65 mg BID achieved significant tumor shrinkage (the maximum target lesion decreased by 18.4%). Twelve patients receiving 85 mg BID, 14 d-on/14 d-off, showed a DCR of 25.0%, and the median PFS was 1.8 months (90% CI, 0.82–3.75) (Figure S1). The efficacy data and swimming plots are shown in Table 4 and Fig. 3, respectively.

**Table 4:** Tumor response assessed by investigators (RECIST 1.1)

	**GFH018** **5 mg BID** **14d-on/14d-off** **(*N* = 4)** ***n* (%)**	**GFH018** **10 mg BID** **14d-on/14d-off** **(*N* = 3)** ***n* (%)**	**GFH018** **20 mg BID** **14d-on/14d-off** **(*N* = 4)** ***n* (%)**	**GFH018** **30 mg BID** **14d-on/14d-off** **(*N* = 7)** ***n* (%)**	**GFH018** **40 mg BID** **14d-on/14d-off** **(*N* = 4)** ***n* (%)**	**GFH018** **50 mg BID** **14d-on/14d-off** **(*N* = 4)** ***n* (%)**	**GFH018** **65 mg BID** **14d-on/14d-off** **(*N* = 6)** ***n* (%)**	**GFH018** **85 mg BID** **7d-on/7d-off** **(*N* = 6)** ***n* (%)**	**GFH018** **85 mg BID** **14d-on/14d-off** **(*N* = 12)** ***n* (%)**	**Total** **(*N* = 50)** ***n* (%)**
**BOR**										
CR	0	0	0	0	0	0	0	0	0	0
PR	0	0	0	0	0	0	0	0	0	0
SD	0	1 (33.3%)	1 (25.0%)	1 (14.3%)	0	1 (25.0%)	1 (16.7%)	1 (16.7%)	3 (25.0%)	9 (18.0%)
PD	2 (50.0%)	2 (66.7%)	1 (25.0%)	3 (42.9%)	3 (75.0%)	1 (25.0%)	5 (83.3%)	2 (33.3%)	4 (33.3%)	23 (46.0%)
NE	0	0	1 (25.0%)^e^	0	0	0	0	0	0	1 (2.0%)^e^
Non-CR/Non-PD^a^	0	0	0	0	0	0	0	0	0	0
NA^b^	2 (50.0%)	0	1 (25.0%)	3 (42.9%)	1 (25.0%)	2 (50.0%)	0	3 (50.0%)	5 (41.7%)	17 (34.0%)
ORR (90%CI)^c^	0	0	0	0	0	0	0	0	0	0
DCR (90%CI)^d^	0	1 (33.3%) (0.84%, 90.57%)	1 (25.0%) (0.63%, 80.59%)	1 (14.3%) (0.36%, 57.87%)	0	1 (25.0%) (0.63%, 80.59%)	1 (16.7%) (0.42%, 64.12%)	1 (16.7%) (0.42%, 64.12%)	3 (25.0%) (5.49%, 57.19%)	9 (18.0%) (8.58%, 31.44%)

Data are shown as *n* (%) unless otherwise specified. Responses were assessed in accordance with Response Evaluation Criteria in Solid Tumors version 1.1

*Abbreviations: BOR* best overall response, *CR* complete response, *DCR* disease control rate, *NE* not estimable, *NA* not available, *ORR* objective response rate, *PD* progressive disease, *PR* partial response, *SD* stable disease

^a^Persistence of 1 or more nontarget lesion(s) and/or maintenance of tumor marker level above the normal limits

^b^No postbaseline assessments after the start date

^c^Defined as the proportion of patients with CR and PR

^d^Defined as the proportion of patients with CR, PR, and SD

^e^SD of insufficient duration (<6 weeks after the start date without further evaluable tumor assessment)

### Biomarker assessment

Baseline serum TGF-β1 data were obtained from 49 patients. The median baseline TGF-β1 level was 27881 pg/mL (range: 10442–58205) in patients who achieved SD and 29213 pg/mL (range: 8645–61434) in patients with progressive disease (Figure S2). This indicated that baseline serum TGF-β1 levels were not associated with clinical efficacy. On-treatment serum TGF-β1 levels were also measured. There was no clear upward or downward trend observed for TGF-β1 levels in either the SD or PD group (Figure S3). pSMAD2 in PBMCs was originally designated as a pharmacodynamic marker of TGF-β pathway inhibition. However, quality control issues associated with the assay preclude data interpretation.

### RP2D determination

The RP2D was determined to be 85 mg BID, 14 d-on/14 d-off based on the safety, PK, and efficacy data. There was no obvious correlation between the incidences of TRAEs and dosages. GFH018 PK-PD analysis in mouse H22 models showed that the efficacy was observed with approximately 50% inhibition of pSMAD3 at a concentration reaching 125 ng/ml after the 1-h dose. The geometric mean of the Cmin of GFH018 was 189.9 ng/ml at the steady state for 85 mg BID, 14 d-on/14 d-off, indicating that after repeated administration, the concentration of GFH018 could be continuously maintained above the effective concentration from preclinical studies. With respect to safety, we have noted that proteinuria occurred across different dosages (Table S4) although generally mild in severity. It seems that no clear dose-dependency for proteinuria, however, one patient experienced grade 3 proteinuria after GFH018 treatment. Considering the future clinical development strategy, i.e., in combination with immune checkpoint inhibitors (ICIs), there might be increasing risk due to overlap toxicity using GFH018 in combination with ICIs at higher dose levels than 85 mg BID, 14d-on/14d-off. Based on the data totality, 85 mg BID 14 d-on/14 d-off is considered as an appropriate dosing regimen for phase II.

## Discussion

The primary purpose of this open-label, first-in-human, phase I study was to evaluate the safety/tolerability of GFH018 and determine the MTD/RP2D. GFH018 showed a favorable safety profile without any DLTs at doses ranging from 5–85 mg BID and demonstrated modest efficacy as monotherapy in patients with advanced solid tumors. The incidence and types of AEs were similar between the doses, with no significant safety signals observed. Regardless of causality, the incidence of G3 TEAEs was 26.0%, while only 6% were judged to be GFH018-related. Liver enzyme increased and anemia were common TRAEs in this study, and most were mild or moderate, which is consistent with other drugs in the same class [11].

Proteinuria was one of the common TRAEs in this study, with most being mild. Only one patient experienced G3-related proteinuria. In addition, no clinically significant changes in serum creatinine levels or estimated glomerular filtration rate indicated renal dysfunction. This is consistent with a study on another SMI, YL-13027, which also reported a high incidence of proteinuria (22.2%) [14]. Evidence shows that TGF-β1 upregulation induces renal extracellular matrix production and glomerular hypertrophy, which correlate with the degree of proteinuria [15]. Blocking TGF-βRI may increase the level of TGF-β1, which potentially contributes to proteinuria. However, conclusions regarding the pleiotropic function of TGF-β are still lacking.

Bleeding is not commonly observed in GFH018 and other SMIs. However, it is frequently observed in mAbs and ligand traps targeting this pathway [16, 17]. Recent studies have revealed the involvement of TGF-β signaling in vascular biology and dysfunction. It plays a role in regulating vascular homeostasis and endothelial cell (EC) activation by differentially activating two type I receptors, TGF-βRI (ALK5) and ALK1 [18–21]. Neutralizing all TGF-β isoforms might block both the ALK1 and ALK5 signaling pathways simultaneously, inhibiting downstream Smad phosphorylation and thus interfering with EC migration, proliferation, and tube formation and influencing vascular formation or reconstruction both physically and pathologically [22].

No special concerns related to skin toxicity were raised for GFH018 as a single agent, which may differ from the results of past studies on other competitors targeting the TGF-β pathway [23]. Several researchers have noted that aberrant TGF-β signaling affects rapid cutaneous squamous cell carcinoma (cSCC) development and might drive cSCC tumorigenesis in the complicated context of the cellular environment [24–26]. However, some have argued that this may also be influenced by the enrolled population and their living habits [27].

TGF-β expression is upregulated in several cardiovascular diseases [28, 29]. The inhibition of TGF-β may lead to changes in cardiovascular structure, increasing the incidence of bleeding, degeneration, and inflammation in the heart valve [30]. Cardiovascular toxicity has been a major obstacle in clinical developments targeting the TGF-β/SMAD pathways. Toxicology studies have shown that cardiac lesions occur with consecutive regimens of GFH018 and galunsertib [30, 31]. Given the essential functions of the heart, an intermittent dosing regimen (14 d-on/14 d-off) was determined as the primary dose regimen in the clinical development of GFH018, providing an acceptable margin of safety. No significant cardiovascular toxicities were observed during the study. Only a few patients experienced a transient increase in cardiac biomarker levels without any symptoms or signs. In addition, 85 mg BID, 7 d-on/7 d-off, was another feasible regimen for further exploration based on the current safety/tolerability data. However, considering that patients with significant cardiovascular disease were not enrolled and that the duration of GFH018 exposure was relatively short, this conclusion needs to be verified with a larger sample size.

In the present study, only modest efficacy was observed. The absence of predictive biomarkers may pose a challenge in the development of drugs targeting this pathway. Alternative strategies should be explored to identify appropriate populations. Several studies have shown that TGF-β is upregulated in the local environment in human papillomavirus (HPV) infections [32]. Inhibition of TGF-β is believed to improve the response while simultaneously blocking PD-1/L1. This approach has been tested in clinical studies on several HPV infection-related tumors, including cervical cancer and HNSCC [33, 34]. Clinical data have shown that patients with advanced HPV-associated malignancies treated with bintrafusp alfa compare favorably with the historical data of pembrolizumab and nivolumab, with an ORR of 35.6% vs. 24% [34]. Moreover, in another ongoing GFH018 phase Ib/II study in combination with toripalimab, promising efficacy was shown in recurrent/metastatic nasopharyngeal carcinoma (R/M NPC) [35], most of which was associated with Epstein‒Barr virus. In fact, elevated serum TGF-β1 levels have been reported in NPC patients with advanced-stage and relapsing tumors [36].

The PK profile of GFH018 showed an observed terminal elimination half-life ranging from 3.11 to 8.60 h for a single dose and from 2.25 to 4.09 h for multiple doses, supporting BID dosing. The T_max_ and t_1/2_ were comparable between the cohorts, indicating similar absorption and elimination characteristics. The geometric CV% of exposure (C_max_ and AUC) at the steady state was 7.3–168.9%, indicating large intersubject variability. The exposure to 85 mg BID 7 d-on/7 d-off was slightly lower than that to 65 mg BID 14 d-on/14 d-off and 85 mg BID 14 d-on/14 d-off, which may be due to the small sample size or large intersubject variability. Overall, exposure to GFH018 increased in an approximately proportional manner in the dose range of 5–85 mg.

Notably, serum specimens were analyzed for TGF-β1 levels in this study. The lack of association of TGF-β1 with the clinical efficacy of GFH018 could be explained by the possibility of excessive TGF-β1 release due to platelet degranulation during the serum preparation process [37]. A recent study indicated that platelet lysis also occurs during plasma preparation and interferes with measured TGF-β1 values [38]. A reliable biomarker for selecting patients who could benefit from the blockade of TGF-β signaling has yet to be identified.

A limitation of this study was its small sample size in each enrolled tumor type. Although multiple tumor types were included in the study, no clear benefit of GFH018 treatment for any specific type could be determined. Additionally, only Chinese patients were enrolled, and the study lacks population diversity and representativity. Nevertheless, to confirm the good safety/tolerability profile of GFH018, further investigation of this agent in combination with immunotherapy and/or chemotherapy is warranted.

## Conclusion

GFH018, in the current dosing regimen, presented a favorable safety profile without cardiovascular toxicity or hemorrhage risk. The modest efficacy of GFH018 as monotherapy was observed in the treatment of patients with advanced solid tumors. GFH018 is currently being tested in combination with immunotherapy and chemotherapy.

### Supplementary Information

**Supplementary Material 1.**

**Supplementary Material 2.**

**Supplementary Material 3.**

**Supplementary Material 4.**

**Supplementary Material 5.**

**Supplementary Material 6.**

**Supplementary Material 7.**

**Supplementary Material 8.**

## Data Availability

Data are available upon reasonable request to corresponding author. Anonymized individual patient data will be shared upon request for research purposes dependent upon the nature of the request, the merit of the proposed research, the availability of the data, and its intended use. The full protocol can be accessed through https://www.clinicaltrials.gov/.

## Abbreviations

ACC: Adenoid cystic carcinoma
AE: Adverse event
AESI: Adverse events of special interest
ALP: Alkaline phosphatase
ALT: Alanine aminotransferase
AST: Aspartate aminotransferase
AUC: Area under the curve
BC: Breast cancer
BID: Twice daily
BNP: Brain natriuretic peptide
BOR: Best overall response
BTC: Biliary tract carcinoma
C1D1: Cycle 1, day 1
Cmax: Maximum concentration
Cmin: Minimum concentration
CR: Complete response
CRC: Colorectal cancer
DA: Duodenal adenocarcinoma
DCR: Disease control rate
DLT: Dose-limiting toxicity
DRC: Dose response curve
EC: Endothelial cell
EC: Endometrial carcinoma
ECOG PS: Eastern Cooperative Oncology Group Performance Status
EoT: End of treatment
FIH: First-in-human
GC: Gastric cancer
GCP: Good clinical practice
GEJC: Gastroesophageal junction cancer
GGT: Gamma-glutamyl transferase
HNSCC: Head and neck squamous cell carcinoma
HPV: Human papillomavirus
Hs-Tn: High-sensitive troponin
ICH: International Council for Harmonization
LDH: Lactate dehydrogenase
LVEF: Left ventricular ejection fraction
MCLN: Metastatic carcinoma of lymoh nodes.
MPM,: Malignant pleural mesothelioma
MTD: Maximum tolerated dose
NA: Not available
NCI-CTCAE: Common Terminology Criteria for Adverse Events v5.0.
NE: Not estimable
NPC: Nasopharyngeal carcinoma
NSCLC: Non-small cell lung cancer
NT-proBNP: N-terminal pro-brain natriuretic peptide
OC: Ovarian cancer
ORR: Objective response rate
PA: Pancreatic cancer
PBMC: Peripheral blood mononuclear cells
PD-1/L1: Programmed death-1 ligand 1
PD: Progressive disease
PFS: Progression-free survival
PK: Pharmacokinetics
PP: Pelvic paraganglioma
PR: Partial response
pSMAD: Phosphorylated SMAD
R_acc_: Accumulation ratio
RDE: Recommended dose for expansion
RECIST: Response evaluation criteria in solid tumors
RP2D: Recommended phase 2 dose
RPC: Renal pelvis carcinoma
SCLC: Small cell lung cancer
SD: Stable disease
SMI: Small molecule inhibitor
TC: Thyroid carcinoma
TEAE: Treatment-emergent adverse event
TGF-β: Transforming growth factor-β
TGF-βR: Transforming growth factor-β receptor
TGF-βRI: Transforming growth factor-β receptor I inhibitor
THYM: Thymic carcinoma
Tmax: Time to reach maximum concentration
TME: Tumor microenvironment
TRAE: Treatment-related adverse event
TTP: Time to progression
UC: Urachus carcinoma
US: Uterus sarcoma
