# Supplementary figures and images for: First-in-human study of GFH018, a small molecule inhibitor of transforming growth factor-β receptor I inhibitor, in patients with advanced solid tumors
Source: BMC Cancer. 2024 Apr 10;24:444. doi: 10.1186/s12885-024-12216-7 (PMC11007962; doi:10.1186/s12885-024-12216-7)

## Slide 1
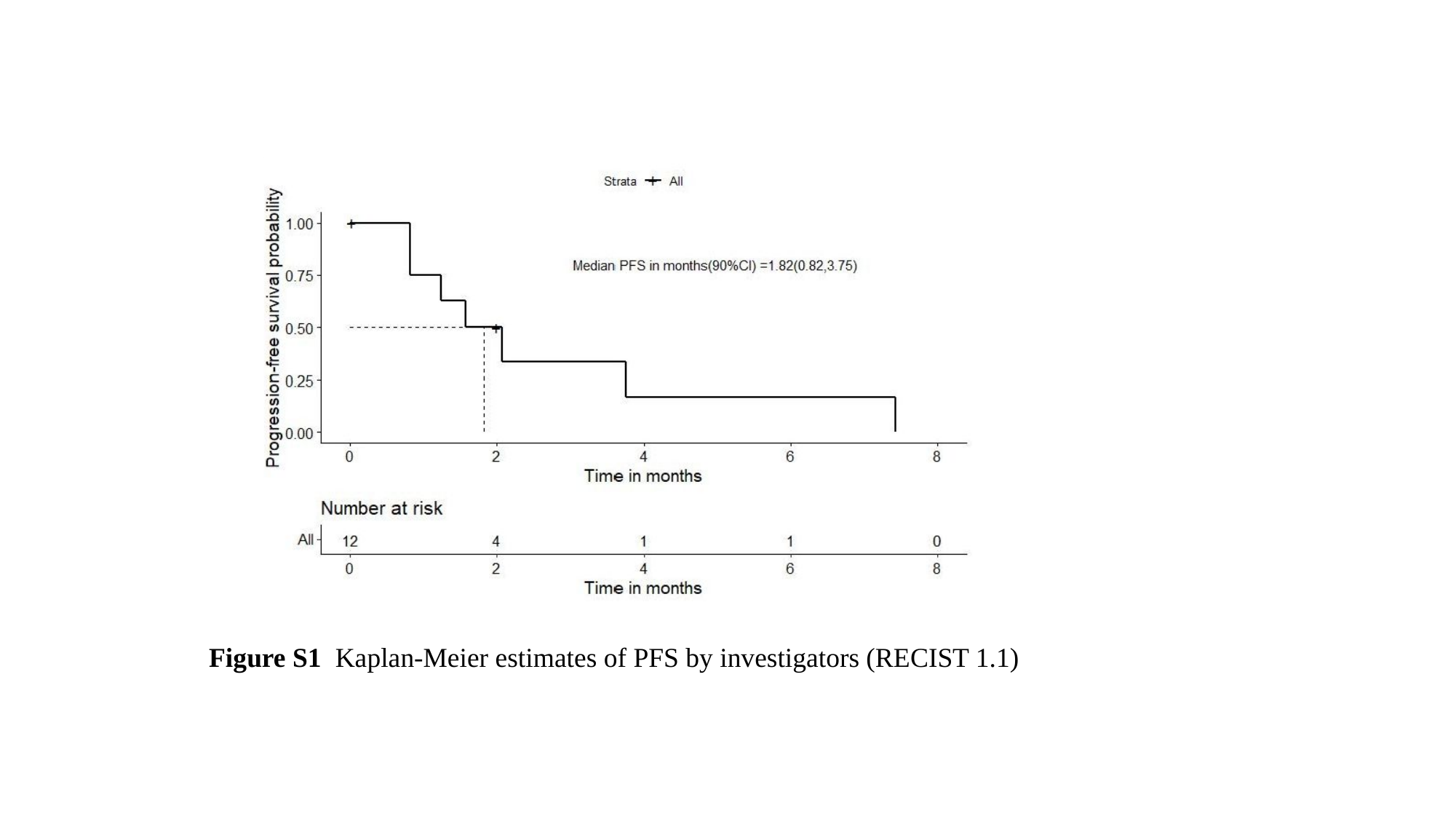

Figure S1 Kaplan-Meier estimates of PFS by investigators (RECIST 1.1)

Supplement: Supplementary file 1 — Supplementary Material 1. [file 12885_2024_12216_MOESM1_ESM.pptx]

## Slide 1
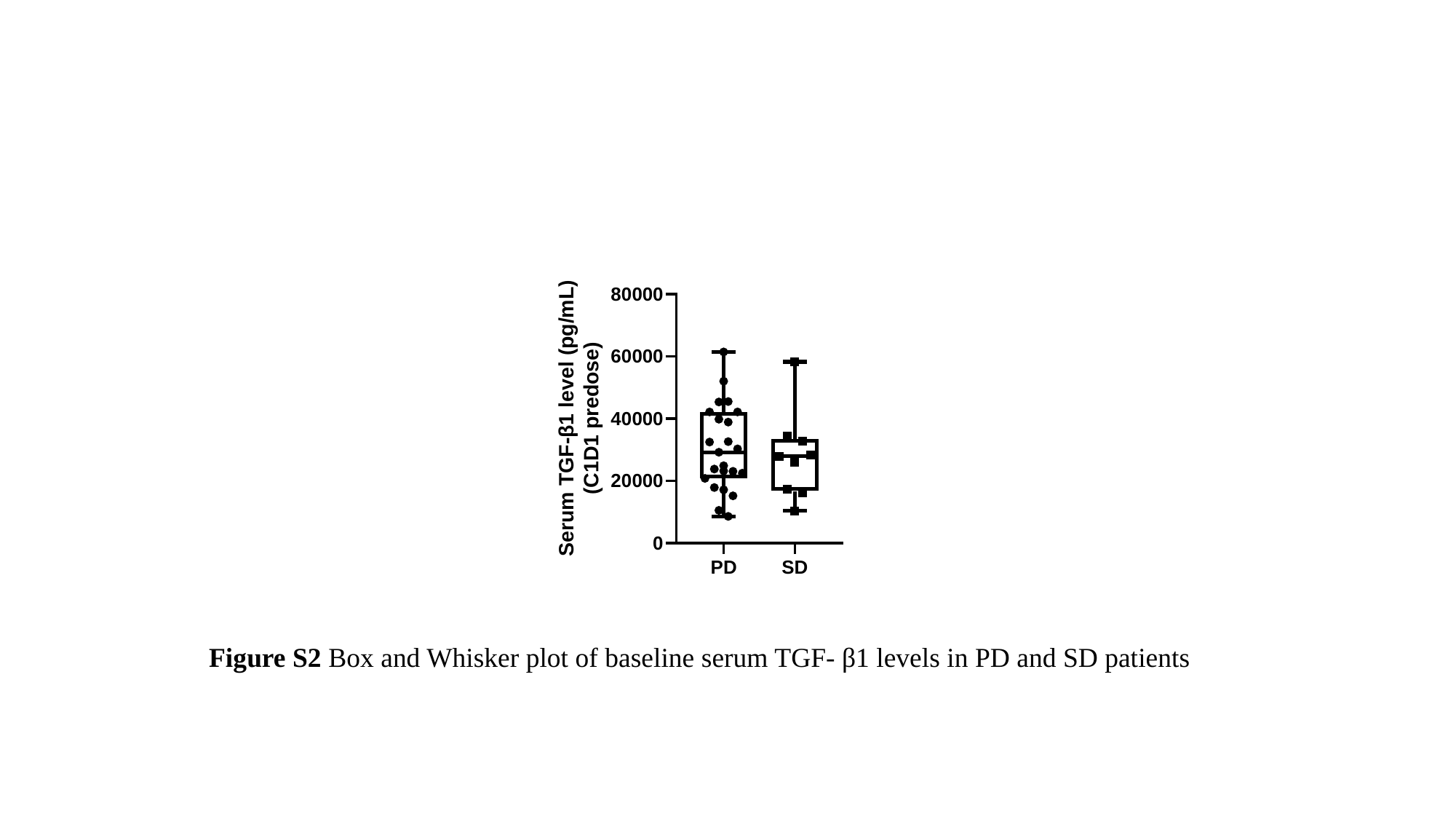

Figure S2 Box and Whisker plot of baseline serum TGF- β1 levels in PD and SD patients

Supplement: Supplementary file 2 — Supplementary Material 2. [file 12885_2024_12216_MOESM2_ESM.pptx]

## Slide 1
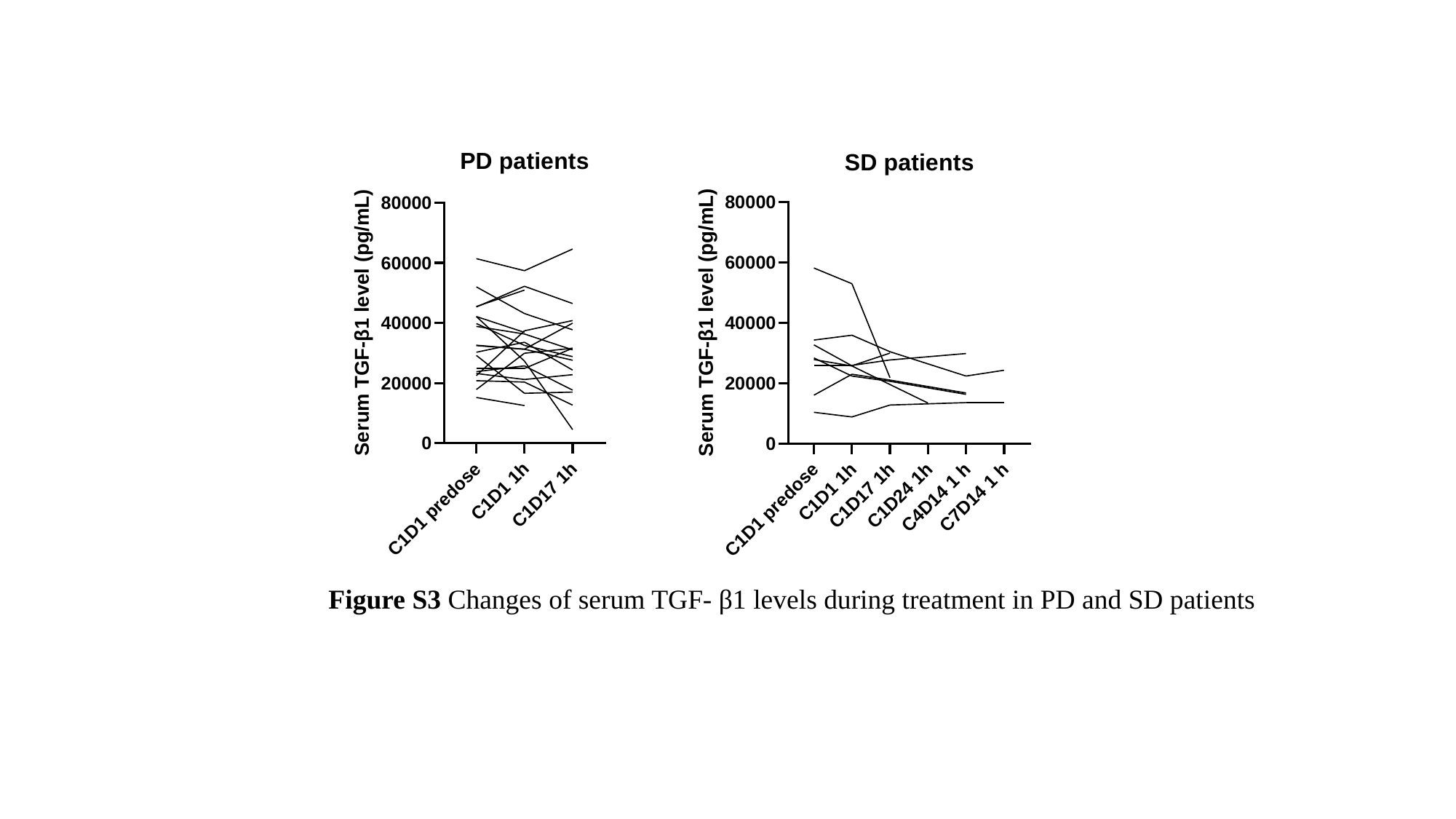

Figure S3 Changes of serum TGF- β1 levels during treatment in PD and SD patients

Supplement: Supplementary file 3 — Supplementary Material 3. [file 12885_2024_12216_MOESM3_ESM.pptx]
