# Supplementary material for: First-in-human study of GFH018, a small molecule inhibitor of transforming growth factor-β receptor I inhibitor, in patients with advanced solid tumors
Source: BMC Cancer. 2024 Apr 10;24:444. doi: 10.1186/s12885-024-12216-7 (PMC11007962; doi:10.1186/s12885-024-12216-7)
Supplement: Supplementary file 4 — Supplementary Material 4. [file 12885_2024_12216_MOESM4_ESM.docx]

# Table S1. Site list

| **Site Number** | **Site Name** | **Location** | **Approval ID number** |
| --- | --- | --- | --- |
| 01 | Shanghai East Hospital, School of Medicine, Tongji University | Shanghai, China | No. EC[2019]061 |
| 03 | Sir Run Run Shaw Hospital, Zhejiang University School of Medicine | Hangzhou, China | 20200609-4 |
| 04 | The Sixth Affiliated Hospital of Sun Yat-Sen University | Guangzhou, China | 2020ZSLYEC-128 |
| 05 | Harbin Medical University Cancer Hospital | Harbin, China | 2021-67 |
| 06 | The First Affiliated Hospital of Bengbu Medical College | Bengbu, China | No. EC[2021]061 |
