# Supplementary material for: First-in-human study of GFH018, a small molecule inhibitor of transforming growth factor-β receptor I inhibitor, in patients with advanced solid tumors
Source: BMC Cancer. 2024 Apr 10;24:444. doi: 10.1186/s12885-024-12216-7 (PMC11007962; doi:10.1186/s12885-024-12216-7)
Supplement: Supplementary file 5 — Supplementary Material 5. [file 12885_2024_12216_MOESM5_ESM.docx]

# Table S2. Treatment-Emergent Adverse Events with Incidence ≥ 10%

| **Preferred term** | **GFH018 5 mg BID 14d-on/14d-off (N = 4) n (%)** | **GFH018 10 mg BID 14d-on/14d-off (N = 3) n (%)** | **GFH018 20 mg BID 14d-on/14d-off (N = 4) n (%)** | **GFH018 30 mg BID 14d-on/14d-off (N = 7) n (%)** | **GFH018 40 mg BID 14d-on/14d-off (N = 4) n (%)** | **GFH018 50 mg BID 14d-on/14d-off (N = 4) n (%)** | **GFH018 65 mg BID 14d-on/14d-off (N = 6) n (%)** | **GFH018 85 mg BID 7d-on/7d-off (N = 6) n (%)** | **GFH018 85 mg BID 14d-on/14d-off (N = 12) n (%)** | **Total (N = 50) n (%)** |
| --- | --- | --- | --- | --- | --- | --- | --- | --- | --- | --- |
| **At least one TEAE** | **4 (100%)** | **3 (100%)** | **4 (100%)** | **7 (100%)** | **4 (100%)** | **4 (100%)** | **5 (83.3%)** | **5 (83.3%)** | **12 (100%)** | **48 (96.0%)** |
| Lymphocyte count decreased | 4 (100%) | 0 | 0 | 1 (14.3%) | 0 | 0 | 0 | 3 (50.0%) | 4 (33.3%) | 12 (24.0%) |
| Urine protein present | 2 (50.0%) | 2 (66.7%) | 2 (50.0%) | 0 | 0 | 1 (25.0%) | 1 (16.7%) | 1 (16.7%) | 1 (8.3%) | 10 (20.0%) |
| AST increased | 0 | 0 | 0 | 3 (42.9%) | 0 | 1 (25.0%) | 2 (33.3%) | 1 (16.7%) | 4 (33.3%) | 11 (22.0%) |
| GGT increased | 1 (25.0%) | 2 (66.7%) | 0 | 1 (14.3%) | 0 | 1 (25.0%) | 1 (16.7%) | 0 | 4 (33.3%) | 10 (20.0%) |
| Bilirubin conjugated increased | 1 (25.0%) | 0 | 1 (25.0%) | 2 (28.6%) | 0 | 0 | 0 | 1 (16.7%) | 3 (25.0%) | 8 (16.0%) |
| ALP increased | 0 | 1 (33.3%) | 1 (25.0%) | 1 (14.3%) | 1 (25.0%) | 1 (25.0%) | 0 | 1 (16.7%) | 2 (16.7%) | 8 (16.0%) |
| White blood cell count decreased | 1 (25.0%) | 0 | 1 (25.0%) | 0 | 0 | 0 | 2 (33.3%) | 1 (16.7%) | 2 (16.7%) | 7 (14.0%) |
| ALT increased | 0 | 1 (33.3%) | 1 (25.0%) | 2 (28.6%) | 0 | 1 (25.0%) | 0 | 0 | 3 (25.0%) | 8 (16.0%) |
| LDH increased | 1 (25.0%) | 1 (33.3%) | 0 | 3 (42.9%) | 1 (25.0%) | 0 | 0 | 0 | 2 (16.7%) | 8 (16.0%) |
| Blood albumin decreased | 2 (50.0%) | 1 (33.3%) | 1 (25.0%) | 0 | 0 | 0 | 0 | 1 (16.7%) | 1 (8.3%) | 6 (12.0%) |
| Platelet count decreased | 1 (25.0%) | 0 | 0 | 1 (14.3%) | 1 (25.0%) | 0 | 1 (16.7%) | 1 (16.7%) | 1 (8.3%) | 6 (12.0%) |
| Blood bilirubin increased | 0 | 0 | 1 (25.0%) | 3 (42.9%) | 0 | 0 | 1 (16.7%) | 0 | 0 | 5 (10.0%) |
| C-reactive protein increased | 2 (50.0%) | 0 | 0 | 0 | 0 | 0 | 0 | 0 | 3 (25.0%) | 5 (10.0%) |
| White blood cells urine positive | 0 | 0 | 1 (25.0%) | 0 | 0 | 0 | 1 (16.7%) | 0 | 3 (25.0%) | 5 (10.0%) |
| Anemia | 0 | 1 (33.3%) | 1 (25.0%) | 3 (42.9%) | 1 (25.0%) | 3 (75.0%) | 3 (50.0%) | 2 (33.3%) | 3 (25.0%) | 17 (34.0%) |
| Abdominal pain | 0 | 0 | 0 | 2 (28.6%) | 0 | 0 | 1 (16.7%) | 2 (33.3%) | 4 (33.3%) | 9 (18.0%) |
| Constipation | 0 | 1 (33.3%) | 0 | 4 (57.1%) | 0 | 0 | 1 (16.7%) | 0 | 2 (16.7%) | 8 (16.0%) |
| Abdominal distention | 0 | 0 | 1 (25.0%) | 2 (28.6%) | 0 | 1 (25.0%) | 0 | 1 (16.7%) | 2 (16.7%) | 7 (14.0%) |
| Hypoalbuminemia | 0 | 0 | 0 | 3 (42.9%) | 0 | 0 | 0 | 1 (16.7%) | 3 (25.0%) | 7 (14.0%) |
| Decreased appetite | 1 (25.0%) | 0 | 0 | 1 (14.3%) | 0 | 1 (25.0%) | 1 (16.7%) | 2 (33.3%) | 0 | 6 (12.0%) |
| Proteinuria | 1 (25.0%) | 0 | 2 (50.0%) | 4 (57.1%) | 0 | 0 | 1 (16.7%) | 1 (16.7%) | 2 (16.7%) | 11 (22.0%) |
| Sinus tachycardia | 2 (50.0%) | 0 | 0 | 1 (14.3%) | 1 (25.0%) | 1 (25.0%) | 2 (33.3%) | 2 (33.3%) | 1 (8.3%) | 10 (20.0%) |
| Asthenia | 0 | 1 (33.3%) | 2 (50.0%) | 2 (28.6%) | 1 (25.0%) | 0 | 0 | 1 (16.7%) | 2 (16.7%) | 9 (18.0%) |

Data are shown as n (%).
