# Supplementary material for: First-in-human study of GFH018, a small molecule inhibitor of transforming growth factor-β receptor I inhibitor, in patients with advanced solid tumors
Source: BMC Cancer. 2024 Apr 10;24:444. doi: 10.1186/s12885-024-12216-7 (PMC11007962; doi:10.1186/s12885-024-12216-7)
Supplement: Supplementary file 6 — Supplementary Material 6. [file 12885_2024_12216_MOESM6_ESM.docx]

**Table S3. Treatment-emergent adverse events with incidences ≥10% in GFH018 85 mg BID 7d-on 7d-off and 14d-on 14d-off**

| **PT  The maximal CTCAE grade** | **GFH018 85 mg BID 7d-on/7d-off (N = 6) n (%)** | **GFH018 85 mg BID 14d-on/14d-off (N = 12) n (%)** | **Total (N = 50) n (%)** |
| --- | --- | --- | --- |
| **At least one TEAE** | **5 (83.3%)** | **12 (100%)** | **48 (96.0%)** |
| AST increased | 1 (16.7%) | 4 (33.3%) | 11 (22.0%) |
| G1 | 1 (16.7%) | 3 (25.0%) | 10 (20.0%) |
| G2 | 0 | 1 (8.3%) | 1 (2.0%) |
| G3 | 0 | 0 | 0 |
| G4 | 0 | 0 | 0 |
| G5 | 0 | 0 | 0 |
| GGT increased | 0 | 4 (33.3%) | 10 (20.0%) |
| G1 | 0 | 3 (25.0%) | 8 (16.0%) |
| G2 | 0 | 1 (8.3%) | 2 (4.0%) |
| G3 | 0 | 0 | 0 |
| G4 | 0 | 0 | 0 |
| G5 | 0 | 0 | 0 |
| ALT increased | 0 | 3 (25.0%) | 8 (16.0%) |
| G1 | 0 | 3 (25.0%) | 8 (16.0%) |
| G2 | 0 | 0 | 0 |
| G3 | 0 | 0 | 0 |
| G4 | 0 | 0 | 0 |
| G5 | 0 | 0 | 0 |
| LDH increased | 0 | 2 (16.7%) | 8 (16.0%) |
| G1 | 0 | 2 (16.7%) | 8 (16.0%) |
| G2 | 0 | 0 | 0 |
| G3 | 0 | 0 | 0 |
| G4 | 0 | 0 | 0 |
| G5 | 0 | 0 | 0 |
| C-reactive protein increased | 0 | 3 (25.0%) | 5 (10.0%) |
| G1 | 0 | 3 (25.0%) | 5 (10.0%) |
| G2 | 0 | 0 | 0 |
| G3 | 0 | 0 | 0 |
| G4 | 0 | 0 | 0 |
| G5 | 0 | 0 | 0 |
| White blood cells urine positive | 0 | 3 (25.0%) | 5 (10.0%) |
| G1 | 0 | 3 (25.0%) | 5 (10.0%) |
| G2 | 0 | 0 | 0 |
| G3 | 0 | 0 | 0 |
| G4 | 0 | 0 | 0 |
| G5 | 0 | 0 | 0 |
| Decreased appetite | 2 (33.3%) | 0 | 6 (12.0%) |
| G1 | 1 (16.7%) | 0 | 4 (8.0%) |
| G2 | 1 (16.7%) | 0 | 2 (4.0%) |
| G3 | 0 | 0 | 0 |
| G4 | 0 | 0 | 0 |
| G5 | 0 | 0 | 0 |

Data are shown as n (%). Adverse events were coded per MedDRA 25.0 and graded according to CTCAE 5.0.

**Abbreviations:** TEAEs, treatment-emergent adverse events. ALT, alanine aminotransferase. AST, aspartate aminotransferase. GGT, γ-glutamyl transpeptidase. LDH, lactate dehydrogenase
