# Supplementary material for: First-in-human study of GFH018, a small molecule inhibitor of transforming growth factor-β receptor I inhibitor, in patients with advanced solid tumors
Source: BMC Cancer. 2024 Apr 10;24:444. doi: 10.1186/s12885-024-12216-7 (PMC11007962; doi:10.1186/s12885-024-12216-7)
Supplement: Supplementary file 7 — Supplementary Material 7. [file 12885_2024_12216_MOESM7_ESM.docx]

**Table S4. Severity of treatment-emergent proteinuria across different GFH018 dosages in the FIH study**

| **Grouped**  **Preferred term** | **GFH018 5 mg BID 14d-on/14d-off (N = 4) n (%)** | **GFH018 10 mg BID 14d-on/14d-off (N = 3) n (%)** | **GFH018 20 mg BID 14d-on/14d-off (N = 4) n (%)** | **GFH018 30 mg BID 14d-on/14d-off (N = 7) n (%)** | **GFH018 40 mg BID 14d-on/14d-off (N = 4) n (%)** | **GFH018 50 mg BID 14d-on/14d-off (N = 4) n (%)** | **GFH018 65 mg BID 14d-on/14d-off (N = 6) n (%)** | **GFH018 85 mg BID 7d-on/7d-off (N = 6) n (%)** | **GFH018 85 mg BID 14d-on/14d-off (N = 12) n (%)** | **Total (N = 50) n (%)** |
| --- | --- | --- | --- | --- | --- | --- | --- | --- | --- | --- |
| Proteinuria* | 3 (75.0) | 2 (66.7) | 4 (100.0) | 4 (57.1) | 0 | 1 (25.0) | 2 (33.3) | 2 (33.3) | 3 (25.0) | 21 (42.0) |
| G1 | 1 (25.0) | 0 | 3 (75.0) | 3 (42.9) | 0 | 1 (25.0) | 1 (16.7) | 2 (33.3) | 1 (8.3) | 12 (24.0) |
| G2 | 2 (50.0) | 2 (66.7) | 1 (25.0) | 1 (14.3) | 0 | 0 | 1 (16.7) | 0 | 1 (8.3) | 8 (16.0) |
| G3 | 0 | 0 | 0 | 0 | 0 | 0 | 0 | 0 | 1 (8.3) | 1 (2.0) |

*Proteinuria was a grouped preferred terms (PTs), presenting PTs including proteinuria and urine protein present coded by MedDRA 25.0.

AEs were graded per CTCAE v5.0.
