# Supplementary material for: First-in-human study of GFH018, a small molecule inhibitor of transforming growth factor-β receptor I inhibitor, in patients with advanced solid tumors
Source: BMC Cancer. 2024 Apr 10;24:444. doi: 10.1186/s12885-024-12216-7 (PMC11007962; doi:10.1186/s12885-024-12216-7)
Supplement: Supplementary file 8 — Supplementary Material 8. [file 12885_2024_12216_MOESM8_ESM.docx]

# Inclusion and Exclusion Criteria

**Inclusion Criteria:**

**Subjects eligible to participate in this study must meet all of the following inclusion criteria:**

1. Voluntary participation in the study and sign the informed consent form.
2. Male or female aged 18–75 years (inclusive).
3. Histologically or cytologically confirmed diagnosis of advanced or metastatic solid tumors with progression on, intolerance to standard therapy, or no suitable standard anticancer therapy available.
4. At least one non-measurable lesion per RECIST 1.1. For patients enrolled in the expansion part, at least one measurable lesion per RECIST 1.1
5. Eastern Cooperative Oncology Group Performance Status (ECOG P.S.) ≤ 1. For patients with liver metastases, Child–Pugh score of 0–7.
6. Life expectancy ≥ 12 weeks.
7. With sufficient organ functions, including:
8. Absolute neutrophil count (ANC) ≥ 1.5 × 10^9^/L, platelet count ≥ 100 × 10^9^/L, hemoglobin ≥ 9 g/dL, without blood transfusion or granulocyte colony-stimulating factor, thrombopoietin, erythropoietin, or other therapies within 14 days prior to hematology tests.
9. Total bilirubin (TBIL) ≤ 1.5 × upper limit of normal (ULN), aspartate aminotransferase (AST) and alanine aminotransferase (ALT) ≤ 2.5 × ULN, alkaline phosphatase (ALP) ≤ 2.5 × ULN; for patients with tumor involvement of the liver, TBIL ≤ 3.0 × ULN, AST and ALT ≤ 5.0 × ULN.
10. Creatinine (Cr) ≤ 1.5 × ULN, or calculated creatinine clearance (CrCl) ≥ 50 mL/min (Cockcroft-Gault formula) if Cr > 1.5 × ULN.
11. International normalized ratio (INR) ≤ 1.5 × ULN.
12. Toxicities left from prior anti-tumor therapy resolved to baseline or CTCAE v5.0 Grade 1 (neurotoxicity or alopecia ≤ Grade 2).
13. For women of childbearing potential (WOCBP) and male subjects with WOCBP partners, agreement to use an effective contraception method from the signing of the informed consent to 90 days after the last administration of the study drug. For WOCBP, negative pregnancy test results within 7 days (inclusive) prior to initiation of the study treatment.
14. Patients or their legal representatives are able to communicate well with the investigators and willing to comply with the protocol and complete the study.

**Exclusion Criteria:**

**Subjects who meet any of the following exclusion criteria are not allowed to enter this clinical study:**

1. With significant cardiovascular diseases:
2. Baseline QT/QTc prolongation (QTcF > 450 ms).
3. Baseline ECG abnormalities of clinical significance
4. Clinically significant cardiovascular diseases within 6 months, eg: myocardial infarction, angina, congestive heart failure, angioplasty, stent implantation, and coronary artery bypass grafting, etc.
5. Abnormal doppler echocardiography confirmed by cardiologist such as left ventricular ejection fraction (LVEF) < 50%,heart valve stenosis, and ≥G2 valve regurgitation.
6. Ascending aorta aneurysm or major artery aneurysm history, or predisposing conditions consistent with the development of aneurysms (for example, family history of aneurysm, Marfan syndrome, evidence of damage to the large vessels of the heart documented by computerized tomography [CT] scan with contrast).
7. Troponin T or I increase of clinical significance.
8. Clinically significant gastrointestinal diseases, such as:

- Intractable hiccup, nausea, and vomiting
- Chronic gastrointestinal disorders: untreated peptic ulcer, Crohn’s Disease, ulcerative colitis, et al.
- Unable to swallow.
- Severe cirrhosis and gastric varicose veins, hepatic encephalopathy
- Active gastrointestinal bleeding.

1. With other severe disease, such as:
2. With definitive neurological or mental disorders, including epilepsy or dementia.
3. Known positive human immunodeficiency virus antibody (HIV-Ab).
4. Active hepatitis B virus infection (positive HBsAg and positive HBV-DNA), or active hepatitis C virus infection (positive HCV-Ab and positive HCV-RNA.
5. With current or history of interstitial pneumonia. Other uncontrolled systemic diseases, such as hypertension and diabetes.
6. Other active infections.
7. Uncontrolled ascites, or pleural effusion clinically.
8. Known active autoimmune diseases or with history of autoimmune diseases that may recur (such as systemic lupus erythematosus, rheumatoid arthritis, inflammatory bowel disease, autoimmune thyroid disease, vasculitis, psoriasis, etc.) or subjects at risk (eg, those who have undergone organ transplantation and require immunosuppressive treatment). However, subjects with well-controlled diabetes mellitus, hypothyroidism requiring only hormone replacement therapy, skin disorders that do not require systemic therapy (such as vitiligo, psoriasis, or alopecia), or who are not expected to relapse in the absence of external triggers.
9. Subjects who need to receive glucocorticoids (prednisone >10 mg/day or equivalent doses of other similar drugs) or other immunosuppressants due to certain conditions within 14 days before treatment initiation.

Note: in the absence of active autoimmune disease, the use of prednisone or equivalent adrenal drugs at a dose of ≤10 mg/day is allowed; subjects are allowed to use topical, ocular, intraarticular, intranasal, and inhaled type of glucocorticoid therapy (extremely low systemic absorption); short-term (≤ 7 days) use of glucocorticoids is permitted for prophylaxis (e.g., contrast media allergy) or for treatment of non-autoimmune conditions (e.g., due to contact allergens) delayed-type hypersensitivity).

1. Unstable brain metastasis. For subjects with brain metastases unintentionally detected during the screening, if they do not cause clinical symptoms and do not require therapeutic intervention, they can be discussed with the sponsor's medical director to decide whether to be enrolled.
2. Pregnant or lactating women.
3. Treatment with chemotherapy, radiotherapy, targeted therapy, endocrine therapy, immunotherapy, or other anti-tumor therapies, or other investigational drugs within 28 days prior to starting the study drug (For mitomycins and nitrosoureas: within 6 weeks. For oral fluorouracils and small molecule targeted drugs: within 2 weeks or 5 half-lives of the drug, whichever is longer).
4. Major surgery (needle biopsy not included) within 4 weeks prior to treatment initiation.
5. Administration of a strong inhibitor or inducer of CYP3A4 within 5 half-life periods or within 2 weeks (whichever is longer), or traditional Chinese medicines within 2 weeks prior to treatment initiation.
6. For subjects enrolled in the expansion part, diagnosis of other malignant tumors within 3 years prior to starting the study drug, except for cured in situ cervical carcinoma and skin basal cell carcinoma.
7. Other conditions judged by the investigator as inappropriate for participation in the study.
